# HPLC-DAD Analysis and *In-Vitro* Property of Polyphenols Extracts from (*Solanum Aethiopium)* Fruits on α -Amylase, α -Glucosidase and Angiotensin - 1- Converting Enzyme Activities

**Published:** 2014-12

**Authors:** E. E Nwanna, E. O Ibukun, G. Oboh, A. O. Ademosun, A. A. Boligon, M. Athayde

**Affiliations:** 1 Department of Biochemistry, Federal University of Technology, P.M.B 704, Akure, 34001 Nigeria;; 2 Program of Post-Graduation in Pharmaceutical Sciences, Federal University of Santa Maria, Campus Camobi, CEP 97105-900, Brazil

**Keywords:** *Solanum aethiopium* species, polyphenols, diabetes, hypertension, vitamin C

## Abstract

**AIM:**

Garden egg (*Solanum aethiopium*) is an edible fruits vegetable with  different species.This study investigated characterisation and the effect of the phenolics extracts from *S. aethiopium* species with enzymes linked with type -2-diabetes (α-amylase and α-glucosidase) and hypertension [Angiotensin-1-converting enzyme (ACE)].

**METHODS:**

Fresh samples of the 5 species of the garden egg namely, [*Solanum gilo* (PW), *Solanum torvum* (TWS), *Solanum kumba* (PGR), *Solanum incanum* (GSB), and *Solanum indicum* (WSB)] were oven-dried at 50°C and milled into flour. The aqueous extracts were prepared (1:50 w/v). The phenolic contents (total phenol and total flavonoid), vitamin C and 1,1-diphenyl–2-picrylhydrazyl (DPPH), the antioxidant activities of the extracts were evaluated. The ability of the extracts to inhibit diabetes enzymes in rat pancreas as well as the inhibition of angiotensin-1-converting (ACE) enzyme in lungs homogenates *in vitro* were investigated. Furthermore, the fruits polyphenols were identified and quantified using HPLC-DAD.

**RESULTS:**

The phenolic contents ranged from 2.70-3.76 mgGAE/g, while there were no significant (*P*>0.05) differences in their flavonoid content and ability to reduce Fe^3+^ to Fe^2+^. The vitamin C contents of the species ranged from 4.01-6.52 mg/ml. The extracts scavenged DPPH in a dose dependent manner with the IC_50_ values ranging from 3.23-4.20 mg/ml. Furthermore, the extracts showed strong inhibition of α-glucosidase, mild inhibition of α-amylase and strong inhibition of ACE activities.

**CONCLUSION:**

This study showed that the inhibition of the key enzymes relevant to type-2 diabetes and hypertension could be part of the mechanisms by which garden egg manage/prevent the degenerative conditions.

## INTRODUCTION

Hyperglycemia is a condition associated with diabetes mellitus and is linked to most diabetes complications as their primary cause. Hyperglycemia is a condition of abnormal rise in plasma glucose level, and in type-2-diabetes is a result of insulin resistance (1). Prolonged hyperglycemia leads to increased generation of reactive oxygen species (ROS) and alteration of endogenous antioxidants. Postprandial hyperglycemia could induce the non-enzymatic glycosylation of various proteins and biomolecules; resulting in the development of chronic complications. Therefore, control of postprandial plasma glucose levels is critical in the early treatment or management of diabetes mellitus, in particular type-2 diabetes, and in reducing chronic vascular complications (2). Inhibition of enzymes involved in the digestion of polysaccharides, such as α-amylase and α-glucosidase, is one of the therapeutic approaches for managing or controlling hyperglycemia (3).

Previous reports have been published on established enzyme inhibitors such as acarbose, miglitol, voglibose, nojirimycin and 1-deoxynojirimycin and their favorable effects on blood glucose levels after food uptake (4). This attribute could be due to inhibition of saccharide assimilation, through inhibiting starch breakdown. With the reduced amount of amylase available for the breakdown, complex polysaccharides have a better chance of travelling through the gastrointestinal tract (GIT) without being assimilated, and are eventually excreted from the body instead of being converted into storage fat. The side effects associated with the use of drugs such as abdominal distension, bloating and flatulene with its cost (5) this necessitates the search for inhibitors from natural sources with strong α-glucosidase, but mild α-amylase activities. One of the long-term complications of type-2-diabetes mellitus is hypertension. Angiotensin –I- converting enzyme (ACE) (EC 3.4.15.1) plays an important physiological role in regulating blood pressure (6). ACE belongs to the class of zinc proteases and is expressed in the vascular endothelial lining of human lungs. ACE is a dipeptidyl carboxypeptidase that catalyzes the conversion of angiotensin I (decapeptide) to angiotensinII (octapeptide), it inactivates the antihypertensive vasodilator (bradykinin) and increases blood pressure (7). Drug development effort has been directed toward excluding unwanted side-effects (8). Inhibition of the angiotensin - 1- converting enzyme is established as one modern therapeutic principle in the treatment of hypertension.

Screening for anti-hypertensive effects in traditional plants has been performed over many years and several animal studies have been carried out (9). Millions of people in developing nations, including Nigerians, have resorted to the use of plants sources to treat or manage their ailments; this could be due to the high cost of orthodox health care or as a result of the global shift towards the use of natural sources, rather than synthetic drugs (10).

*Solanum aethiopicum* “Gardenegg” or “Scarlet eggplant” is an edible vegetable crop belonging to the family *Solanaceae*. The family is one of the largest and the most important families of vegetable which are essentially tropical in origin. Phytochemical analysis on *Solanum aethiopicum,* has been reported (11, 12).

Reports on the pharmacological activity of these garden eggs have been reported on its antiulcer (12).This plant has been regarded as an underutilised crop possible because there is scanty knowledge and scientific information about it (13). The garden egg species have been in use in traditional medicine systems for the management of type-2 diabetes and the National Diabetes Education Programme of National Institute of Health, the Mayo Clinic and American Diabetes Association (14, 15) has also recommended garden egg as choice plant in the management of the degenerative condition (16).There are different species of these garden egg (*Solanum aethiopicum*), endowed in Nigeria. Studies have been done recently on some species (17) in continuation of the research studies were undertaken on five common species in order to rank them based on their anti- diabetic/ anti-hypertensive activities since there are limited information on their mechanism of action, also to quantify and characterized the poly- phenols present which could give more information that contributes to the ranking of garden egg. Therefore, it is expedient to investigate the *in-vitro* inhibitory effect of water extractable phytochemicals from these species of garden egg(*Solanum aethiopium*) on α-amylase, α-glucosidase and angiotensin - 1- converting enzyme (ACE) activities.

## MATERIALS AND METHODS

### Sample Collection

Fresh samples of 5 species of Garden egg (*Solanum aethiopum) *commonly consumed in Nigeria [*Solanum gilo* (PW), *Solanum torvum* (TWS), *Solanum kumba* (PGR), *Solanum incanum* (GSB), and *Solanum* *indicum* (WSB)] were purchased from Erekesan main market at Akure,Ondo State, Nigeria. The identification of the samples was carried out at the Crop, Soil, and Pest management (CSP) Department of the Federal University of Technology, Akure, Nigeria. All the samples was oven-dried at 50°C and milled into powder,and was stored in an airtight plastic container. All the chemicals used were of analytical grade, and distilled water was used for analyses.

### Chemicals and equipment

Folin-Ciocalteu’s phenol reagent, gallic acid and anhydrous sodium carbonate used were products of Fluka (Buchs, Switzerland). Quercetin and DPPH (2,2-diphenyl-1picrylhydrazyl), Ascorbic acid and starch were products of Merck (Darmstadt, Germany), Iron chloride, ACE, porcine pancreatic α-amylase (EC 3.2.1.1) and α-glucosidase (EC 3.2.1.20) were products of Sigma-Aldrich (USA). Iron (III) chloride 6-hydrate and trichloroacetic acid Fisher products. All other chemicals used were purchased from Rovet Scientific Limited, Benin City, Edo State, Nigeria. The distilled water used was obtained from the Chemistry Department at Federal University of Technology, Akure. Optical absorbance was measured with a UV-Visible spectrophotometer (Model 6305; Jenway, Barloworld Scientific, Dunmow, United Kingdom). Methanol, acetic, gallic, caffeic ellagic acid, kaempferol and chlorogenic acids were purchased from Merck (Darmstadt, Germany). Catechin, ,epicatechin, quercetin, quercitrin,isoquercitrin and rutin were acquired from Sigma Chemical Co. (St. Louis, MO, USA). High performance liquid chromatography (HPLC-DAD) was performed with a Shimadzu Prominence Auto Sampler (SIL-20A) HPLC system (Shimadzu, Kyoto, Japan), equipped with Shimadzu LC-20AT reciprocating pumps connected to a DGU 20A5 degasser with a CBM 20A integrator, SPD-M20A diode array detector and LC solution 1.22 SP1 software.

### Sample Preparation

The aqueous extracts were prepared (1:50 w/v). 1g of each of milled samples was soaked in 50ml distilled water for 24hrs. The mixture was filtered and the filtrate was centrifuged to obtain a clear supernatant which was subsequently used for the various assays (18).

### Total phenol determination

The total phenol content of the sample was determined by adding 0.5 ml of the sample extract to an equal volume of water, 2.5 ml 10% Folin-Ciocalteau reagent (v/v) and 2.0 ml of 7.5% sodium carbonate was subsequently added. The reaction mixture was incubated at 45°C for 40 min, and absorbance was measured at 726 nm (JENWAY 6305), Gallic acid was used as the standard phenol (19).

### Determination of total flavonoid content

The total flavonoid content of the extracts was determined using a slightly modified method reported by (20). Briefly, 0.5 ml of aqueous sample was mixed with 0.5 ml methanol, 50 μL of 10% AlCl_3_, 50 μL of 1mol/L potassium acetate and 1.4 ml water, and allowed to incubate at room temperature for 30 min. Thereafter, the absorbance of the reaction mixture was subsequently measured at 415 nm. The total flavonoid content as calculated using quercetin as standard.

### Determination of vitamin C content

Vitamin C content of the garden egg extracts was determined using the method of Benderitter (21). Briefly, 75 µL DNPH (2 g dinitrophenyl hydrazine, 230 mg thiourea and 270 mg CuSO_4_·5H_2_O in 100 ml of 5 M H_2_SO_4_) were added to 500 µL reaction mixture (300 µL of appropriate dilution of the extracts with 100 µL 13.3% trichloroacetic acid (TCA) and water). The reaction mixture was subsequently incubated for 3 hrs 37°C, then 0.5 ml of 65% H_2_SO_4_ (v/v) was added to the medium and the absorbance was measured at 520 nm using a spectrophotometer. The vitamin C content of the extracts was subsequently calculated.

### Ferric reducing power (FRAP)

;The reducing property of the extracts was determined by assessing the ability of the extract to reduce FeCl_3_ solution as described by Oyaizu (22). A 2.5 ml aliquot was mixed with 2.5 ml of 200 mM sodium phosphate buffer (pH 6.6), and 2.5 ml of 1% potassium ferricyanide. The mixture was incubated at 50°C for 20 min, and then 2.5ml of 10% trichloroacetic acid was added. This mixture was centrifuged at 650 rpm for 10 min, thereafter 5 ml of the supernatant was mixed with an equal volume of water and 1 ml of 0.1% ferric chloride. The absorbance was measured at 700 nm against a reagent blank. A higher absorbance indicates a higher reducing power.

### 1,1-diphenyl-2-picrylhydrazyl (DPPH) free radical scavenging ability

The free radical scavenging ability of the extracts against DPPH (1,1-diphenyl–2 picrylhydrazyl) free radical was evaluated as described by Gyamfi (23). Appropriate dilution of the extracts was mixed with 1 ml of 0.4 mM methanolic solution containing DPPH radicals. The mixture was left in the dark for 30 min and the absorbance was measured at 516 nm. The DPPH free radical scavenging ability was subsequently calculated with respect to the reference, which contained all the reagents without the test sample.

### α-Amylase inhibition assay

The aqueous extracts volume (500 μl) and 500 μl of 0.02M sodium phosphate buffer (pH 6.9 with 0.006 M NaCl) containing Hog pancreatic α-amylase (EC 3.2.1.1) (0.5 mg/ml) were incubated at 25°C for 10 minutes. Then, 500 μl of 1% starch solution in 0.02 M sodium phosphate buffer (pH 6.9 with 0.006 M NaCl) was added to each tube. The reaction mixtures was incubated at 25°C for 10 minutes and stopped with 1.0 ml of dinitrosalicylic acid colour reagent. Thereafter, the mixture was incubated in a boiling water bath for 5 minutes, and cooled to room temperature. The reaction mixture was then diluted by adding 10 ml of distilled water, and absorbance measured at 540 nm using the spectrophotometer (JENWAY 6305). The percentage (%) enzyme inhibitory activity of the aqueous extracts was calculated (24).

### α-Glucosidase inhibition assay

The volume of the aqueous extracts (50 μL) and 100 μl of α-glucosidase solution (1.0 U/ml) in 0.1 M phosphate buffer (pH 6.9) was incubated at 25°C for 10 min. Then, 50 μl of 5 mM p-nitrophenyl-α-D-glucopyranoside solution in 0.1 M phosphate buffer (pH 6.9) was added. The mixtures were incubated at 25°C for 5 min. Then 2ml of Na_2_CO_3_ was added to terminate the reaction before reading the absorbance at 405 nm in the spectrophotometer (JENWAY 6305). The α-glucosidase inhibitory activity was expressed as percentage inhibition. The percentage (%) enzyme inhibitory activity of the aqueous extracts was calculated (25).

### Angiotensin-I-converting enzyme (ACE) inhibition assay

The volume of the aqueous extract (50 μl) and ACE solution (50 μl, 4 mU) were incubated at 37°C for 15 min. The enzymatic reaction was initiated by adding 150 μl of 8.33 mM of the substrate Bz–Gly–His–Leu in 125 mM Tris– HCl buffer (pH 8.3) to the mixture. After incubation for 30 min at 37ºC, the reaction was arrested by adding 250 μl of 1M HCl. The cleaved Gly–His bond and the Bz–Gly produced by the reaction was extracted with 1.5 ml ethyl acetate. Thereafter the mixture was centrifuged to separate the ethyl acetate layer; then 1 ml of the ethyl acetate layer was transferred to a clean test tube and evaporated. The residue was redissolved in distilled water and its absorbance was measured at 228 nm using the spectrophotometer (JENWAY 6305). The percentage (%) enzyme inhibitory activity of the aqueous extracts was calculated. (26).

### Quantification of compounds by HPLC-coupled with diode array detection (DAD)

Reverse phase chromatographic analyses were carried out under gradient conditions using C_18_ column (4.6 mm × 150 mm) packed with 5 μm diameter particles; the mobile phase was water containing 2% acetic acid (A) and methanol (B), and the composition gradient was: 5% of B until 2 min and changed to obtain 25%, 40%, 50%, 60%, 70% and 100% B at 10, 20, 30, 40, 50 and 60 min, respectively, following the method described by Amaral (27) with slight modifications. All the samples were analyzed at a concentration of 20 mg/mL. The presence of eleven antioxidants compounds was investigated, namely, gallic acid, caffeic acid, ellagic acid, chlorogenic acid, kaempferol, catechin, epicatechin, quercetin, quercitrin,isoquercitrin and rutin. Identification of these compounds was performed by comparing their retention time and UV absorption spectrum with those of the commercial standards. The flow rate was 0.7 ml/min, injection volume 40 μl and the wavelength were 254 nm for gallic acid, 280 nm catechin and epicatechin, 327 nm for caffeic, ellagic acid and chlorogenic acids, and 365 nm for quercetin, quercitrin: isoquercitrin, kaempferol and rutin. The samples and mobile phase were filtered through 0.45 μm membrane filter (Millipore) and then degassed by ultrasonic bath prior to use. Stock solutions of standards references were prepared in the HPLC mobile phase at a concentration range of 0.020–0.200 mg/ml for quercetin, quercitrin isoquercitrin, kaempferol rutin, catechin and epicatechin; and 0.050–0.250 mg/ml for chlorogenic, ellagic acid, caffeic and gallic acids. The chromatography peaks were confirmed by comparing its retention time with those of reference standards and by DAD spectra (200 to 500 nm). Calibration curve for gallic acid: Y = 12563x + 1381.4 (r=0.9991); caffeic acid: Y = 12738x + 1527.2 (r=0.9999); ellagic acid: Y = 13084x + 1256.7 (r=0.9997); chlorogenic acid: Y = 10972x + 1375.4 (r=0.9998); rutin: Y = 11782 + 1460.3 (r=0.9997); quercetin: Y = 12895x + 1342.5 (r=0.9993); quercitrin: Y = 11753x + 1439.6 (r=0.9997); isoquercitrin: Y = 10982x + 1242.1 (r=0.9996); kaempferol: Y = 13940 + 1173.9 (r=0.9989);catechin: Y = 12194x + 1407.9 (r=0.9995) and epicatechin: Y = 14176x + 1305.4 (r=0.9991). All chromatography operations were carried out at ambient temperature and in triplicate. The limit of detection (LOD) and limit of quantification (LOQ) were calculated based on the standard deviation of the responses and the slope using three independent analytical curves, as defined (28) LOD and LOQ were calculated as 3.3 and 10 σ/S, respectively, where σ is the standard deviation of the response and S is the slope of the calibration curve.

### Determination of IC_50_

In order to determine the IC_50_ (the concentration of extracts required to inhibit 50% of the enzyme activity) values, the percentage of enzyme inhibition of the garden egg extracts was plotted against the extracts at various concentrations for α-amylase, α-glucosidase and angiotensin-1- converting enzyme. DPPH free radical Scavenging ability inhibition was also determine. The IC_50_ was then calculated using linear regression.

### Data analysis

The results of the three replicate experiments were pooled and expressed as mean ± standard deviation (SD). Analysis of variance and the least significant difference test were carried out Significance was accepted (*P*≤0.05) (29), followed by multiply comparison test (Tukey test) different letters differ by (*p*<0.01) for HPLC DAD characterizations of the phenolics.

## RESULTS

**Figure 1:**
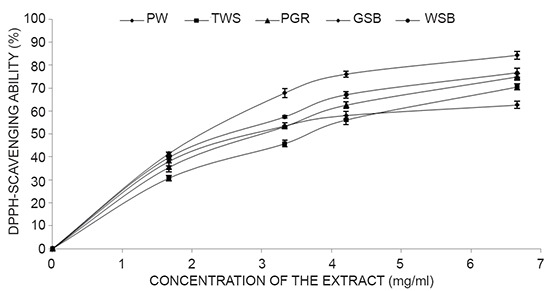
Free radical scavenging ability of the aqueous extract of Garden egg species. Key: *Solanum gilo* (PW), *Solanum torvum* (TWS), *Solanum kumba* (PGR), *Solanum incanum* (GSB), and *Solanum indicum* (WSB). Values represent means ± standard deviation of triplicate readings.

Total phenolic content reported as gallic acid equivalent ofthe garden egg varied between 2.70 mg GAE/gram-3.76 mg GAE/gram while the total flavonoid contents ranged from 1.22-1.60 mg QE/ gram (Table 1). Vitamin C content ranged from 4.01-6.52mg/ml as shown from the studies (Table 1). The ferric reducing antioxidant capacity (FRAP) of the extract reported as ascorbic acid equivalents is a measure of the ability of the phenolic extracts to reduce Fe(III) to Fe(II) a measure of their antioxidant properties. However, all the extracts showed good ferric reducing antioxidant property 5.72 mg/g - 6.89 mg/g (Table 1). All the studied species showed appreciable free radical scavenging activities in a dose-dependent manner in the range of (3.23-4.20 mg/ml) Figure 1 and Table 1 from the IC_50_ values (concentration of extract causing 50% inhibitory activity) of the extract.The lower the value the higher the inhibitory activities.

**Table 1:** Total phenol, Total flavonoid, Vitamin C content, Reducing power and IC_50_ DPPH of the aqueous extracts of *Solanum aethiopicum* Species

SAMPLES	Total Phenols (mgGAE/g)	Totals Flavonoids (mgQE/g)	Vitamin C (mg/ml)	Reducing Power (mg/g)	EC50of DPPH (mg/ml)

GSB	3.76 ± 0.11^a^	1.50 ± 0.17^a^	5.82 ± 0.15^b^	6.89 ± 0.12^a^	3.23 ± 0.06^a^
WSB	3.76 ± 0.11^a^	1.72 ± 0.09^a^	6.52 ± 0.11^a^	5.98 ± 0.11^b^	3.62 ± 0.23^a^
PGR	3.44 ± 0.21^a^	1.22 ± 0.25^a^	5.82 ± 0.15^b^	5.79 ± 0.04^b^	3.80 ± 0.31^a^
TWS	2.70 ± 0.07^b^	1.60 ± 0.17^a^	4.01 ± 0.15^b^	5.90 ± 0.43^b^	4.18 ± 0.10^b^
PW	2.83 ± 0.19^b^	1.22 ± 0.25^a^	5.55 ± 0.41^b^	5.72 ± 0.35^b^	4.20 ± 0.14^b^

*Solanum gilo* (PW), *Solanum torvum* (TWS), *Solanum kumba* (PGR), *Solanum incanum* (GSB), and *Solanum indicum* (WSB). Data represent means of triplicate determinations. Values with the same letter along the same column are not significantly different (*P*<0.05).

**Figure 2:**
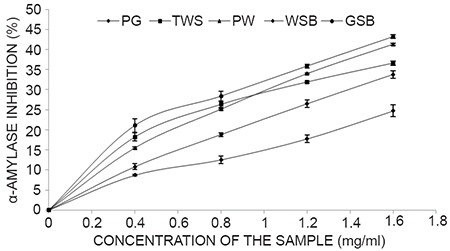
α-Amylase inhibitory activity of aqueous extract of Garden egg species. Key: *Solanum gilo* (PW), *Solanum torvum* (TWS), *Solanum kumba* (PGR), *Solanum incanum* (GSB), and *Solanum indicum* (WSB). Values represent means ± standard deviation of triplicate readings.

**Figure 3:**
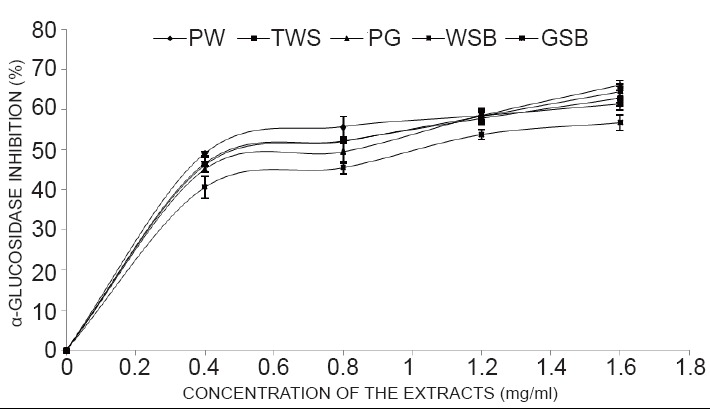
α-Glucosidase inhibitory activity of aqueous extract of Garden egg species. Key: *Solanum gilo* (PW), *Solanum torvum* (TWS), *Solanum kumba* (PGR), *Solanum incanum* (GSB), and *Solanum indicum* (WSB). Values represent means ± standard deviation of triplicate readings.

**Figure 4:**
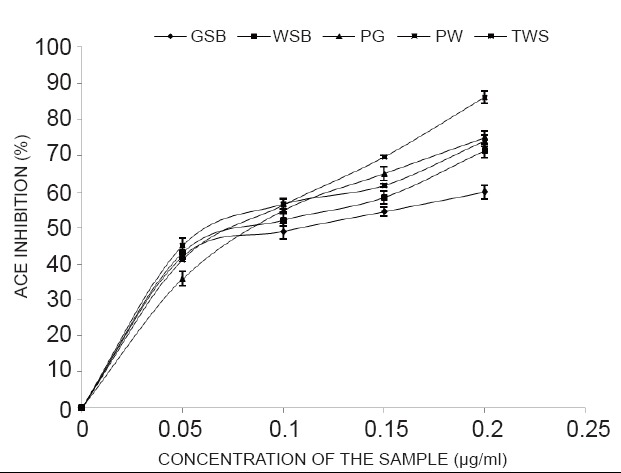
Angiotensin-1-converting enzyme (ACE) inhibitory activity of the aqueous extract of garden egg. Key: *Solanum gilo* (PW), *Solanum torvum* (TWS), *Solanum kumba* (PGR), *Solanum incanum* (GSB), and *Solanum indicum* (WSB). Values represent means ± standard deviation of triplicate readings.

The results from Table 2, as indicated from the IC_50_ revealed strong inhibition of α-glucosidase (1.02-1.12 mg/ml),mild inhibition of α-amylase (1.67-3.23mg/ml) enzymes linked to diabetes..The lower the value the higher the inhibitory activities.However, there was strong inhibition of angiotensin - 1- Converting enzyme (ACE) enzyme activity linked to hypertension of all the garden egg species extract in a dose-dependent manner as shown [106-138 μg/ml] in which TWS showed the highest inhibition of ACE (Figure 2, 3 and 4).

**Table 2:** IC_50_ of enzymes inhibitory activities

SAMPLES	α-amylase (mg/ml)	α-glucosidase (mg/ml)	Angiotensin-1-Converting enzyme (ACE) (μg/ml)

GSB	1.67 ± 0.01^a^	1.02 ± 0.02b	138 ± 0.02b
WSB	2.28 ± 0.04b	1.03 ± 0.01b	123 ± 0.02b
PGR	1.80 ± 0.12^a^	1.03 ± 0.01b	117 ± 0.01^a^
TWS	1.92 ± 0.21^a^	1.04 ± 0.01b	106 ± 0.01^a^
PW	3.23 ± 0.12b	1.12 ± 0.01^a^	117 ± 0.01^a^

*Solanum gilo* (PW), *Solanum torvum* (TWS), *Solanum kumba* (PGR), *Solanum incanum* (GSB), and *Solanum indicum* (WSB).Data represent means of triplicate determinations. Values with the same letter along the same column are not significantly different (*P*<0.05).

**Figure 5:**
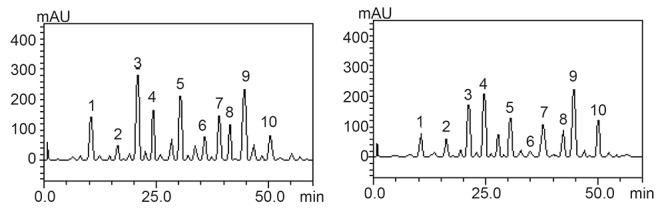
Representative high performance liquid chromatography profile of PGR and GSB Gallic acid (peak 1), catechin (peak 2), chlorogenic acid (peak 3), caffeic acid (peak 4), ellagic acid (peak 5), epicatechin (peak 6), rutin (peak 7), quercitrin (peak 8), quercetin (peak 9) and kaempferol (peak 10).

**Figure 6:**
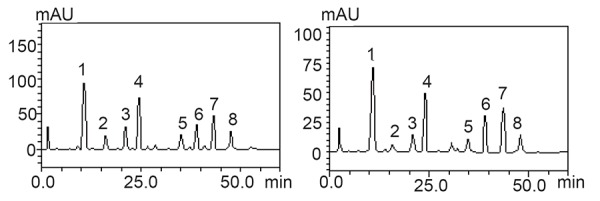
Representative high performance liquid chromatography profile of TWS and WSB extract. Gallic acid (peak 1), catechin (peak2), chlorogenic acid (peak 3), caffeic acid (peak 4), epicatechin (peak 5), rutin (peak 6), isoquercitrin (peak 7) and quercetin (peak 8).

**Figure 7:**
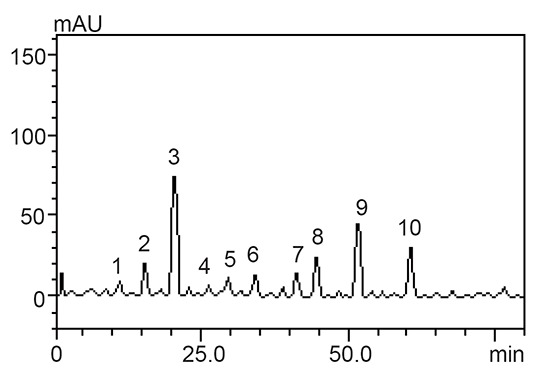
Representative high performance liquid chromatography profile of PW. Gallic acid (peak 1), catechin (peak 2), chlorogenic acid (peak 3), caffeic acid (peak 4), ellagic acid (peak 5), epicatechin (peak 6), rutin (peak 7), quercitrin (peak 8), quercetin (peak 9) and kaempferol (peak 10).

Chromatograhic fingerprinting of the extracts using reverse - phase HPLC-DAD is presented in Table 3 and Figure 5, 6 and 7. The samples revealed the presence of some phenolic acids and flavonoids and their derivatives. Amongst these phenolic constituents gallic acid, rutin, quercetrin, isoquercetrin kaempferol, catechin, epicatechin, were present as the major phenolic compounds while ellagic acid, caffeic and chlorogenic acid were identified and quantified through their retention times [10 min – 60 min] for the 1^st^ peak to the 10^th^ peak and UV spectra.The quantification ranking also revealed that TWS had the highest gallic acid, caffeic acid, epicatechin, rutin, isoquercitrin followed by PGR,GSB and PW has all the analysed phenols except isoquercitrin while WSB has no ellagic acid, quercitrin, kaempferol this results gotten correspond with its anti-hypentensive ability.

**Table 3:** Phenolic composition of garden egg species using HPLC-DAD analysis

Samples	PGR	GSB	TWS	WSB	PW

Compounds	mg/g	mg/g	mg/g	mg/g	mg/g
Gallic acid	17.36 ± 0.02^a^	6.28 ± 0.01^a^	50.34 ± 0.01^a^	34.90 ± 0.01^a^	2.73 ± 0.01^a^
Catechin	4.25 ± 0.01^b^	6.13 ± 0.02^a^	12.67 ± 0.02^b^	4.17 ± 0.03^b^	5.39 ± 0.01^b^
Chlorogenic acid	30.81 ± 0.01^c^	18.65 ± 0.01^b^	20.71 ± 0.01^c^	10.63 ± 0.01^c^	20.67 ± 0.03^c^
Caffeic acid	19.76 ± 0.03^d^	23.79 ± 0.02^c^	39.46 ± 0.03^d^	27.21 ± 0.03^d^	2.15 ± 0.01^d^
Ellagic acid	25.13 ± 0.03^e^	13.26 ± 0.01^d^	ND	ND	2.93 ± 0.02^a^
Epicatechin	6.32 ± 0.01^f^	2.51 ± 0.02^e^	14.53 ± 0.02^b^	8.54 ± 0.02^c^	3.12 ± 0.03^a^
Rutin	16.89 ± 0.03^c^	12.38 ± 0.02^d^	22.18 ± 0.01^c^	21.78 ± 0.03^e^	4.98 ± 0.01^b^
Quercitrin	12.45 ± 0.01^g^	6.45 ± 0.02^a^	ND	ND	6.17 ± 0.03^b^
Quercetin	26.47 ± 0.02^e^	27.05 ± 0.03^f^	14.92 ± 0.03^b^	9.15 ± 0.03^c^	12.58 ± 0.01^e^
Kaempferol	7.08 ± 0.03^f^	12.68 ± 0.01^d^	ND	ND	10.92 ± 0.03^f^
Isoquercitrin	ND	ND	28.36 ± 0.01^e^	23.41 ± 0.01^e^	ND

Results are expressed as mean ± standard deviations (SD) of three determinations. Averages followed by different letters differ by Tukey test at *p*<0.01. ND, NOT DETECTED.

## DISCUSSION

The positive health effects of phenolic phytochemicals are linked to their ability to counter the negative effects of reactive oxygen species generated during cellular energy metabolism (30). Flavonoids are also known as a class of widely distributed phytochemicals with antioxidant activities (31). *Solanum aethiopicum* species are rich sources of phenolic phytochemicals which is in agreement with the studies done by (32). Ascorbic acid is a good reducing agent and exhibits its antioxidant activities by electron donation (33). Moreso, it is widely distributed in plant cells where it plays many crucial roles in growth and metabolism. As a potent antioxidant, ascorbic acid has the capacity to eliminate several different reactive oxygen species, keeps the membrane-bound antioxidant α-tocopherol in the reduced state, acts as a cofactor maintaining the activity of a number of enzymes (34). *Solanum* *indicum* had the highest ranking for vitamic C content among the species tested in this study. The ability of the extracts to reduce Fe (III) to Fe (II), is a measure of their antioxidant properties and this could be liked to their phenolic. The link between free radical formation and the development and complications of diabetes has been established (35, 36). Phenolics and flavonoids have been shown to be effective in preventing diabetes in animal models (35). The radical scavenging ability of the garden egg and its ferric reducing antioxidant property agrees with previous findings by (37).

The current therapeutic approach to the management if diabetes and its related complications is the inhibition of starch metabolizing enzymes such as α-amylase and α-glucosidase activities as this will control the catabolism of starch into glucose and there will moderation of blood glucose level (38). This present study on *Solanum aethiopicum* garden egg extracts showed a concentration dependent inhibition of α-amylase and α-glucosidase activities. Furthermore, the IC_50_ values revealed that the extracts showed a stronger inhibition of α-glucosidase activity than α-amylase activity and this is therapeutically important in preventing some of the side effects associated with the use of synthetic drugs on diabetes enzymes (39). However, *Solanum incacum* had the strongest inhibition while *Solanum gilo* had the least inhibition of the enzymes’ activities. Inhibition of angiotensin -1- converting enzymes (ACE) activity is currently use in the management of hypertension which is the one of the complications associated with type-2-diabetes (40). The garden egg extracts inhibited ACE activity *in-vitro* in a concentration–dependent manner. The results from the enzymes inhibition followed the trend of the phenolics distribution among the species. However, it was discovered that *Solanum torvum* which had the least phenolic and vitamin C contents, and antioxidant activities but exhibited better ACE inhibition followed by *Solanum Kumba*. Gallic acid, catechin, chlorogenic acid, caffeic acid, epicatechin, quercetinare the phenolics compound that were identified in all the species while there was variations with the present of ellagic acid, kaemferol, isoquercitrin, quercitin. Epidemiological studies have found that a positive association between the consumption of foods containing these phenolics and a reduced risk of developing several disorders such as cardiovascular diseases, antidiabetic antimicrobial, inflammatory, neurogenicals activities etc. (41-43). This may be as a result of the individual or synergistic activities of its phenols and flavonoids as reported by (44) on different Iranian *Allium* species. The results of this study show that phenolics-rich extracts from these *S. aethiopicum* species could be exploited for potential use in pharmaceutical formulations for preventive medicine in the management of type-2 diabetes and hypertension.

## CONCLUSION

Our studies strongly suggest that *Solanum aeithiopicum* garden eggs can be promising sources of potential nutraceuticals/functional food. The differences in the phenolics/flavonoids content could explain the differences in the observed bioactivities based on the HPLC-DAD analysis, however, *in-vivo* studies is recommended.
